# Flexural Strength of Concrete Beam Reinforced with CFRP Bars: A Review

**DOI:** 10.3390/ma15031144

**Published:** 2022-02-01

**Authors:** Mohd Basri Che Bakar, Raizal Saifulnaz Muhammad Rashid, Mugahed Amran, Mohd Saleh Jaafar, Nikolai Ivanovicn Vatin, Roman Fediuk

**Affiliations:** 1 Department of Civil Engineering, Universiti Putra Malaysia, Serdang 43400, Malaysia; raizal@upm.edu.my (R.S.M.R.); msj@upm.edu.my (M.S.J.); 2 Public Works Department Malaysia, Kuala Lumpur 50582, Malaysia; 3 Department of Civil Engineering, College of Engineering, Prince Sattam Bin Abdulaziz University, Alkharj 16273, Saudi Arabia; m.amran@psau.edu.sa; 4 Department of Civil Engineering, Faculty of Engineering and IT, Amran University, Amran 9677, Yemen; 5 Peter the Great St. Petersburg Polytechnic University, 195251 St. Petersburg, Russia; vatin@mail.ru (N.I.V.); fedyuk.rs@dvfu.ru (R.F.); 6 Polytechnic Institute, Far Eastern Federal University, 690922 Vladivostok, Russia

**Keywords:** carbon fiber, CFRP bars, CFRP reinforcement, flexural strength

## Abstract

Conventional reinforced concrete (RC) structures are commonly associated with the corrosion of steel reinforcement. The application of carbon fiber reinforced polymer (CFRP) bars as flexural reinforcement has become a new promising option. This paper presents a state-of-the art flexural strength on concrete beams reinforced with CFRP bars. Concrete compressive and CFRP bar tensile strain, reinforcement ratio, types of surface treatment on CFRP bar and concrete compressive strength were identified as aspects of behavior. Significant findings in the literature had manifested all aspects of behavior that were affecting the flexural strength, deflections and crack characteristics of CFRP RC beams. In addition, the experimental result on 98 specimens of CFRP RC beams from the literature show that ACI 440.1R-15 and CSA S806-12 standards underestimate the ultimate flexural moment capacity of CFRP RC beams. On the other hand, Kara and Ashour predictions are more accurate with the experimental values. Moreover, hotspot research topics were also highlighted for further considerations in future studies.

## 1. Introduction

Corrosion of steel reinforcement is a common threat to the reinforced concrete (RC) structures that are particularly exposed to the marine, aggressive and highly corrosive environment. They are susceptible to accelerating the carbonation process, and chloride and sulphate attacks that eventually contribute to the corrosion of steel reinforcement [1,2,3]. Corroded reinforcement subsequently affects the serviceability performance and long-term durability of RC structures themselves [4,5]. In addition, it requires extensive and expensive maintenance to minimize the effect of steel reinforcement corrosion [5]. Therefore, the development of a new technique in order to overcome the reinforcement corrosion issues is imminent. As a result, the application of carbon fiber reinforced polymer (CFRP) bars as flexural reinforcement has become a promising option in the past 20 years [6].

It is well-established that CFRP bars have gained enormous popularity for their relevant material characteristics in terms of the excellent tensile strength (e.g., 600 MPa to 3690 MPa), high strength to weight ratio (e.g., 10 to 15 times greater than steel bars), noncorrosive and nonmagnetic materials [6,7,8,9,10,11,12]. Moreover, CFRP bars are the most resistant to chemical environments, including acid, alkaline and saline solutions [13,14,15]. With these significant advantages, CFRP bars have attracted great interest to replace steel bars in RC structures [7,16]. Additionally, CFRP RC structures offer an ultimate solution for problems of electromagnetic interference, intentionally required for the Magnetic Resonance Imaging (MRI) rooms in the hospitals and research institutes [7,17].

Most of the researchers and design codes encourage the design of CFRP RC beams as over-reinforced since it leads to a less catastrophic failure of CFRP ruptures [5,6,7,18,19,20,21]. Moreover, it exhibits excessive deflections and wider cracks at serviceability limit states (SLS) due to a lower modulus of elasticity than steel bars [20,22,23]. Hence, the performance of CFRP RC beams is generally controlled at SLS [24]. Concerning this, the effects of concrete compressive strength on over-reinforced CFRP RC beams is paramount. Another primary factor affecting the flexural strength of CFRP RC beams is the bond characteristics between CFRP bars and concrete. The bond strength between CFRP bars and concrete contribute a significant role in achieving the ultimate capacity of the CFRP RC beam [8]. Henceforth, flexural strength is predicted by ignoring the bond behavior and assumed to have a perfect bond [6,18,19,25]. In addition, there is no unified model available that can be applied to generate the required minimum bond strength of CFRP bars as flexural reinforcement. Thus, it is necessary to overview the effects of surface treatment on the flexural performances of CFRP RC beams [26,27,28,29,30].

Until now, there are limited numbers of research that have been conducted to investigate the effectiveness of CFRP bars as flexural reinforcement. Hence, the understanding of the beam’s flexural behavior remains deficient. Moreover, significant variation of CFRP bars available in the market with a difference in material characteristics might as well contribute to the knowledge gap. Therefore, this paper intends to highlight the current state-of-the-art reports related to the flexural strength of RC beams reinforced with CFRP bars. To take full advantage of the knowledge and data mined from the literature, this review study focuses on several fundamental issues related to flexural strength. This primarily emphasis the material properties and behavior for concrete and CFRP bars—a normal practice in the experimental works and flexural behavior of the RC beam reinforced with CFRP bars. Experimental results are compared with the international design standards, and hotspot research topics for future investigations are also discussed in this study.

## 2. Material Properties and Behavior for Concrete and CFRP Bar

**Figure 1:**
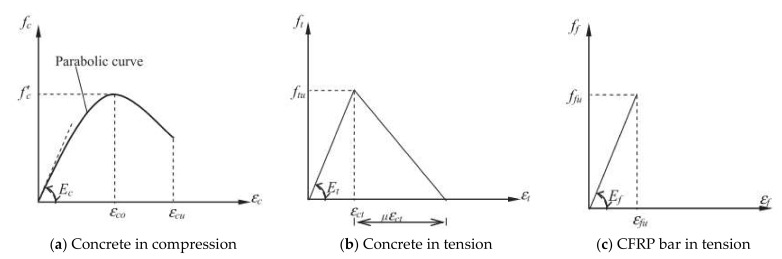
Stress-strain relationship for concrete and CFRP bar (Reprinted with permission from ref. [25], 2022, Elsevier).

Figure 1a,b present the stress–strain relationships of concrete in compression and tension, respectively. The concrete compressive behavior in the stress–strain curve can be calculated by the numerical technique and written as Equation (1) [31]. This expression relates the stress, *f_c_* and the strain at that particular stress, *ε_c_*.

(1) $$f_{c}=f_{c}^{\prime }\left(\frac{2\varepsilon_{c}}{\varepsilon_{c0}}-{\left(\frac{\varepsilon_{c}}{\varepsilon_{c0}}\right)}^{2}\right);\varepsilon_{c}<\varepsilon_{cu}$$

where; $f_{c}$ and $\varepsilon_{c}$ are the compressive stress (MPa) and strain in concrete, respectively; $f_{c}^{\prime }$ is the maximum cylinder compressive strength of concrete (MPa); $\varepsilon_{c0}$ is the strain at the maximum stress $f_{c}^{\prime }$ (commonly taken as 0.002); and, $\varepsilon_{cu}$ is the ultimate compressive strain in concrete. The concrete behavior in compression has controlled the failure for an over-reinforced CFRP RC beam.

Figure 1b depicts a typical stress–strain curve of concrete in tension. The curve exhibits two discrete branches consisting of an essentially linear relationship (i.e., before cracking) and a concave descending curve (i.e., after cracking). The concrete in tension may be expressed as below [31]:

(2) $$f_{t}=E_{c}\varepsilon_{t};\varepsilon_{t}\leq \varepsilon_{cr}$$

(3) $$f_{t}=f_{cr}{\left(\frac{\varepsilon_{cr}}{\varepsilon_{t}}\right)}^{0.4};\varepsilon_{t}>\varepsilon_{cr}$$

where $f_{t}$ and $\varepsilon_{t}$ are the tensile stress (MPa) and strain in concrete, respectively; $E_{c}$ is the concrete modulus of elasticity (MPa); $\varepsilon_{cr}$ is the concrete cracking strain (taken as 0.00008); and, $f_{cr}$ is the concrete cracking stress (MPa) (taken as $0.31\sqrt{f_{c}^{\prime }}$). However, the concrete strength in tensile is ignored in calculating the nominal flexural strength, *M_n_* of CFRP RC beam [6,18].

On the other hand, the stress–strain relationship of CFRP bar is linear elastic up to failure, as shown in Figure 1c. Normally, CFRP bar fails in tension in a sudden and catastrophic manner, as well as accompanied by a loud sound. This behavior is demanding more attention in designing under-reinforced CFRP RC beams. The tensile strength of CFRP bar, *f_f_* is given by [6]:

(4) $$f_{f}=E_{f}\varepsilon_{f};\varepsilon_{f}\leq \varepsilon_{fu}$$

where $f_{f}$ and $\varepsilon_{f}$ are the CFRP bar tensile stress (MPa) and strain, respectively; $E_{f}$ is the CFRP bar tensile modulus of elasticity (MPa); and, $\varepsilon_{fu}$ is the CFRP bar ultimate strain.

## 3. Flexural Behavior of CFRP RC Beam

### 3.1. Beam Specimen and Test Procedures

**Figure 2:**
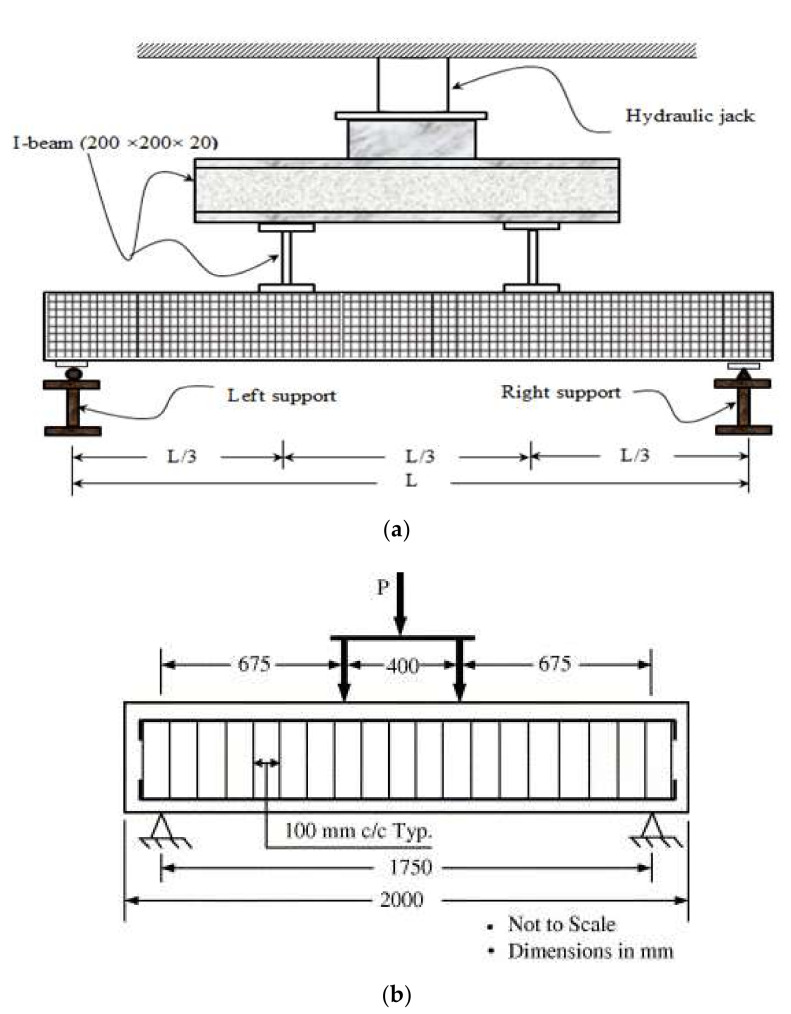
(**a**) Flexural test set up with a single point load (Reprinted with permission from ref. [42], 2022, Elsevier) [42] and (**b**) Flexural test set up with a pair of point load (Reprinted with permission from ref. [22], 2022, Elsevier): Flexural tests set up.

Typical rectangular CFRP RC beam specimens were commonly tested under a flexural test. The beam specimens were frequently subjected either to a single or pair of point load, as shown in Figure 2. The beam specimens were simply supported with hinge support at one end and roller support at the other end. The test parameters were varied, including the concrete compressive strength and the material characteristics of CFRP bar, particularly on different types of surface treatment and reinforcement ratios [17,22,32,33,34,35,36,37,38,39,40,41]. Strain gauges were internally installed on the CFRP bars’ reinforcement to record its tensile strains throughout the test period. They were bonded using adhesive and were fully waterproofed before pouring the concrete into the formwork. Prior to conducting the flexural test with a minimum concrete age of 28 days, strain gauges were also installed on the surface at the top of the beam specimen to measure concrete compressive strains, whilst, beam deflections were measured using a linear variable differential transformer (LVDT) located at the mid-span beam. In addition, horizontal LVDTs may be placed at both sides of the beam specimen in order to measure the slip between CFRP bars and concrete during the flexural test [8]. A data-acquisition module was monitored by a computer to record the readings of the strain gauges, LVDTs and load cells.

The flexural test was subdivided into three stages: (i) prior to crack; (ii) first flexural crack; and, (iii) specimen failure. The load was gradually increased until the first flexural crack appears. After the first crack occurred, the loading increment was controlled by deflection at a constant range until the specimen failure. Also, crack formation along the sides of the white-painted beam was marked and recorded to trace the crack propagations. Specimen failure was defined as either the CFRP RC beam fails in flexural, or a de-bond of CFRP bar. In any circumstances, shear failure of the CFRP RC beam should be avoided. The applied load was released after the specimen failure and no further data were recorded.

### 3.2. Modes of Failure

It is noteworthy that most CFRP RC beams were designed as over-reinforced sections [17,22,32,33,34,35,36,37,38,39,40,41]. This strength limit state is based on the reinforcement ratio that is greater than the balanced reinforcement ratio. Reinforcement ratio, $\rho_{f}$ and balanced reinforcement ratio, $\rho_{fb}$ can be calculated using Equations (5) and (6), respectively. The terms *α1* and *β1* are determined with Equations (7) and (8) for ACI 440.1R-15 [6] and Equations (9) and (10) for CSA S806-12 [18]. The balanced reinforcement ratio is corresponding to the condition where compressive strain in the extreme concrete fibers achieve its ultimate value of $\varepsilon_{cu}$ = 0.003 [6] or $\varepsilon_{cu}$ = 0.0035 [18] and, simultaneously CFRP bars achieve their ultimate tensile strain. On the other hand, CFRP RC beam is considered an under-reinforced limit state when its reinforcement ratio is less than the balanced reinforcement ratio condition.

(5) $$\rho_{f}=\frac{A_{f}}{bd}$$

where $\rho_{f}$ is the reinforcement ratio; *A_f_* is the area of CFRP bars (mm^2^); *b* is the width of the rectangular cross-section (mm); and, *d* is the distance from extreme compression fiber to centroid of CFRP bars (mm).

(6) $$\rho_{fb}=\alpha_{1}\beta_{1}\frac{f_{c}^{\prime }}{f_{fu}}\frac{E_{f}\varepsilon_{cu}}{E_{f}\varepsilon_{cu}+f_{fu}}$$

where $\rho_{fb}$ is the balanced reinforcement ratio; *f_c_′* is the compressive strength of concrete (MPa); *f_fu_* is the ultimate tensile strength of CFRP bars considering environmental reduction factors (MPa); *E_f_* is the modulus of elasticity of CFRP bars (MPa); and, *ε_cu_* equals to 0.003 [6] or 0.0035 [18] for the ultimate strain considered in concrete. Where $\alpha_{1}$ and *β_1_* can be calculated as follows:

(7) $$\alpha_{1}=0.85$$

(8) $$\beta_{1}=0.85-0.05\left(\frac{f_{c}^{\prime }-28}{7}\right)\geq 0.65$$

(9) $$\alpha_{1}=0.85-0.0015f_{c}^{\prime }\geq 0.67$$

(10) $$\beta_{1}=0.85-0.0025f_{c}^{\prime }\geq 0.67$$

The failure mode for the over-reinforced CFRP RC beam is concrete crushing. Therefore, concrete in an extreme compression zone for this beam is expected to crush and the corresponding moment is recorded as the nominal moment capacity, *M_n_*. At this level, the concrete compressive strain may achieve more than 0.3%. The beam, however, could carry more loads with concrete in the compression zone to continue to crush and spall. Afterwards, the beam finally reaches its ultimate moments, *M_u_* when the applied load subsequently dropped. The ratio of *M_u_/M_n_* is higher than 1.0. This controlling limit strength is so-called compression-controlled [6] where the beam may only exhibit concrete crushing without CFRP bars rupture. However, in the condition of normalized reinforcement ratio, *ρ_f_* /*ρ_fb_* in the range of 1.0 to 1.4, the beam experiences concrete crushing as well as CFRP bars rupture [6].

On the other hand, failure mode for under-reinforced CFRP RC beam is governed by CFRP bars rupture. In the literature, Gravina and Smith [7] and Al-Sunna et al. [43] suggested avoiding designing an under-reinforced CFRP RC beam as the CFRP bars are brittle and prone to sudden tensile failure. The beam has failed catastrophically at mid-span corresponding to its ultimate moment, *M_u_*. Generally, the beam exhibits few cracks and higher crack width. The beam failure may be accompanied by a loud sound when the CFRP bars rupture due to the tensile strain increasing a linearly reach to its ultimate elongation [37].

**Figure 3:**
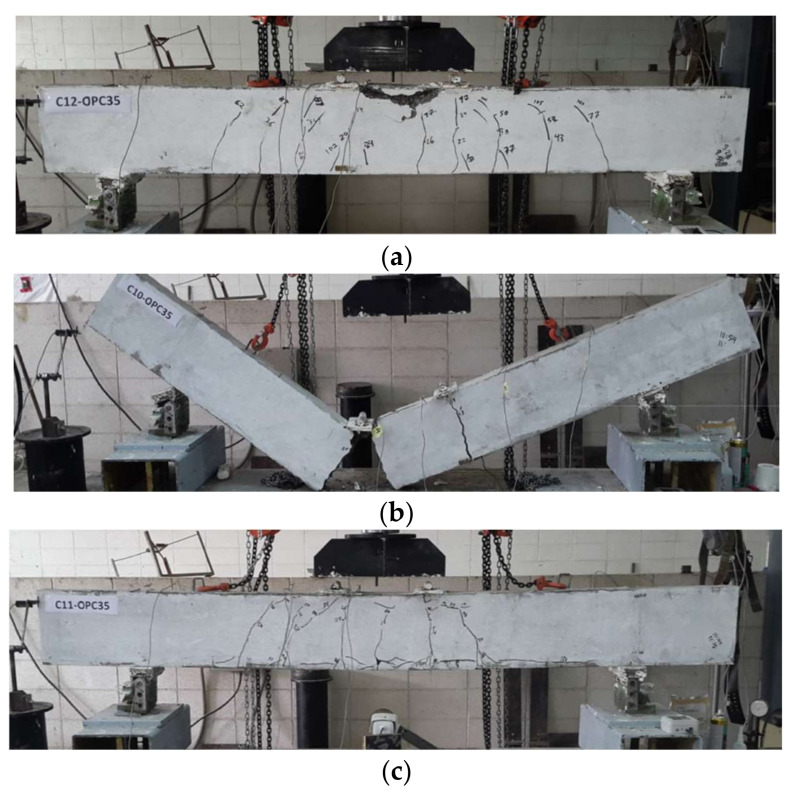
Depicts each mode of failure for CFRP RC beams: (**a**) Concrete crush failure, (**b**) CFRP bars rupture, and (**c**) De-bond failure between CFRP bars and concrete (Reprinted with permission from ref. [17], 2022, Elsevier).

In addition, another failure mode that has to be considered is the de-bond of CFRP bars from surrounding concrete. This type of failure is either due to insufficient bond strength between CFRP bars and concrete or localized bond failure of the CFRP bars between flexural cracks [17]. Recent research indicated that assuring sufficient bond strength provides crucial interfacial shear stress between CFRP bars and concrete [8]. This interfacial shear stress may avoid any unnecessary slippage while transferring flexural stress in the CFRP RC beam tension zone. On the other hand, localized bond failure of the CFRP bars and concrete may initiate at the extreme tensile zone of the beam. The initial flexural cracks observed at concrete cover propagate as horizontal cracks along the tensile CFRP bars reinforcement. This behavior might be attributed to the sudden transfer of localized tensile forces from the cracked concrete to the CFRP bar’s interface [17]. Figure 3 depicts each mode of failure for CFRP RC beams: (a) Concrete crush failure, (b) CFRP bars rupture, and (c) De-bond failure between CFRP bars and concrete.

## 4. Aspect Behavior of Flexural Strength

### 4.1. Concrete Compressive Strain and CFRP Bars Tensile Strain Behavior

**Figure 4:**
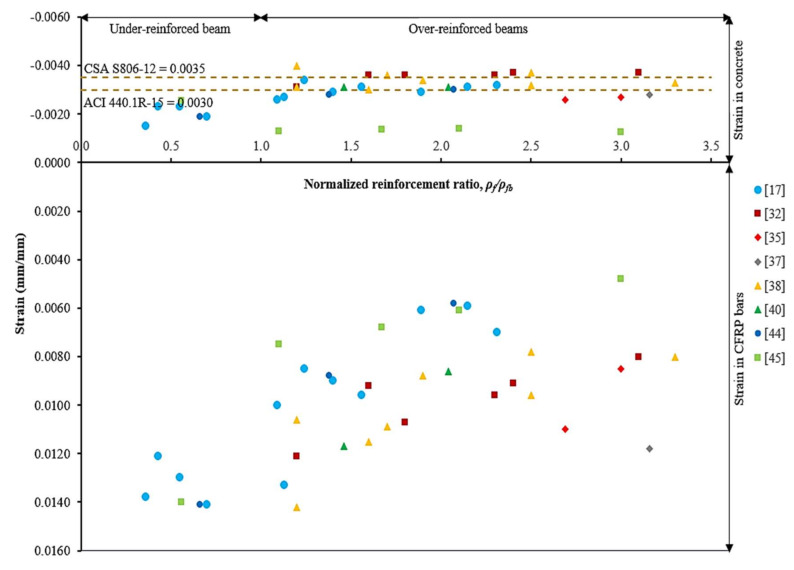
Concrete compressive and CFRP bars’ tensile strain behavior [17,32,35,37,38,40,44,45].

Figure 4 shows the ultimate concrete compressive strain and CFRP bar tensile strain for different reinforcement ratios of CFRP RC beams. The CFRP RC beam strength limit state, with respect to normalized reinforcement ratio, could be easily identified from the graph. Such an example of under-reinforced limit state has indicated that the CFRP bar had achieved its ultimate tensile strength at failure. The maximum tensile strain in CFRP bars was 105% over its ultimate capacity [17,44]. Therefore, this beam had experienced CFRP bar ruptures at failure. For over-reinforced beams in a transition zone (i.e., 1.0 < *ρ_f_*/*ρ_fb_* < 1.4) [6], compressive strains in concrete were in the range of 0.0026 to 0.0040 [17,32,38]. As for CFRP bars, tensile strains were in the range of 0.0100 to 0.0142 [17,32,38] and equivalent to 74.1% and 110.9% of their ultimate capacity. The results have indicated that beams had failed by concrete crushing and may be followed by CFRP bar rupture. The beams were so-called compression-tension controlled failure [6].

Further comparative studies with over-reinforced beams have found that the compressive strains in concrete were between 0.0026 and 0.0040. It can be observed that only 24.2% of beams had recorded concrete compressive strain over 0.0035, whilst, 63.6% were over 0.0030. Therefore, the assumption of a maximum concrete compressive strain of 0.0030 is more reliable in computing the nominal flexural strength. Tensile strains in CFRP bars for over-reinforced beams, however, were recorded in the range of 28.4% to 84.2% of their ultimate capacity. The beams had failed by concrete crushing without CFRP bar ruptures. The over-reinforced beam (i.e., *ρ_f_*/*ρ_fb_* ≥ 1.4) is so-called compression-controlled failure [6].

### 4.2. Effect of Reinforcement Ratio

**Figure 5:**
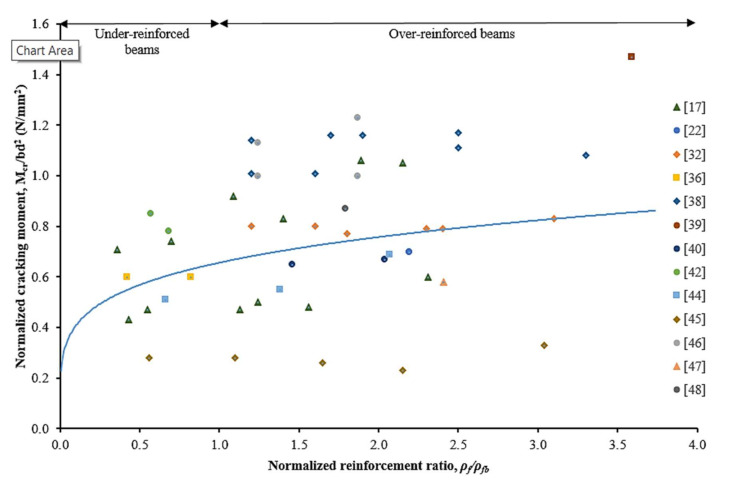
Normalized cracking moment against normalized reinforcement ratio [17,22,32,36,38,39,40,42,44,45,46,47,48].

**Figure 6:**
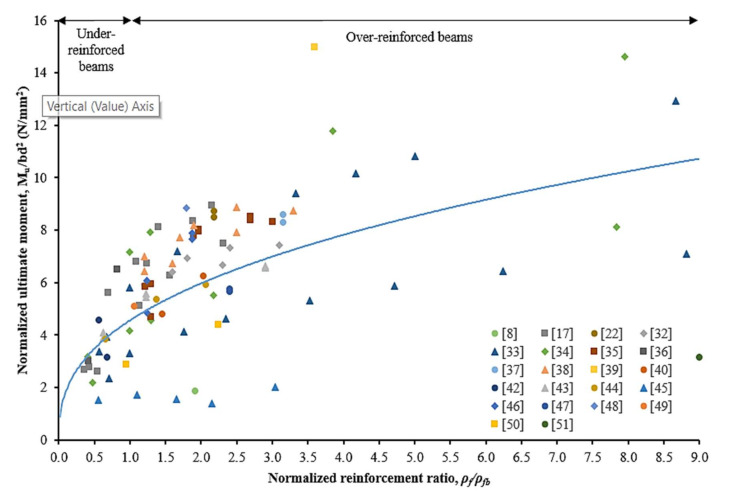
Effects of normalized reinforcement ratio on the normalized ultimate moment [8,17,22,32,33,34,35,36,37,38,39,40,42,43,44,45,46,47,48,49,50,51].

Data mined from literature have been employed to study the effect of increasing the reinforcement ratio of CFRP bars to the beam cross-section. Figure 5 and Figure 6 present the effect of being increased normalized reinforcement ratio to the normalized cracking moment and the ultimate moment of CFRP RC beams, respectively. In general, the reinforcement ratio had affected the behavior and stiffness of the beam specimens, which resulted in both normalized cracking and ultimate moment. It can be clearly observed that the normalized cracking and ultimate moment had been increased by increasing the CFRP reinforcement ratio.

The results on the effect of reinforcement ratio to normalized cracking moment are scattered, as depicted in Figure 5. Nevertheless, over-reinforced beams have had higher cracking moments rather than under-reinforced beams. This behavior can be attributed due to the tensile force being transferred to a higher reinforcement area at the extreme tensile concrete zone. From Figure 6, it can be manifested that the normalized ultimate moment was increased along with the increment of reinforcement ratio. The strength limit state for under-reinforced and over-reinforced beams is identified and marked on the graph. While over-reinforced beams resulted in higher moment capacity, it indicates less significance on the increment rates, particularly at *ρ_f_/ρ_fb_* > 4.0. For the under-reinforced case, the ultimate flexural moment capacity exhibits is more reliant on the increment of reinforcement ratio.

### 4.3. Variation of Concrete Compressive Strength

**Figure 7:**
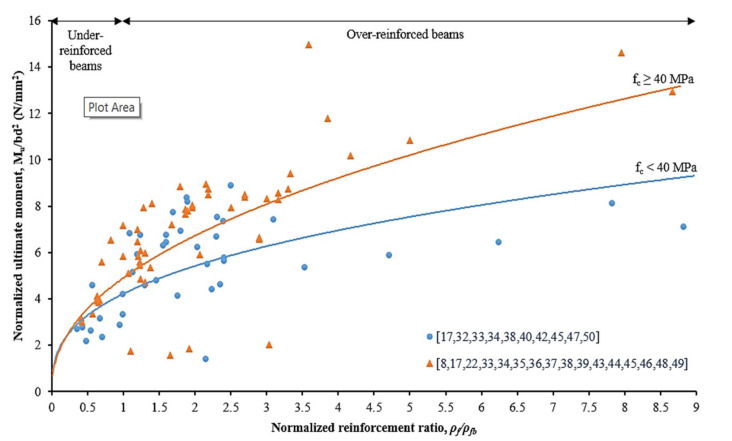
Effects of normalized reinforcement ratio on the ultimate flexural moment capacity with respect to concrete compressive strength [8,17,22,32,33,34,35,36,37,38,39,40,42,43,44,45,46,47,48,49,50].

Figure 7 is plotted for two different concrete compressive strengths, categorized as lower than 40 N/mm^2^ and greater than 40 N/mm^2^. The strength limit state for the under-reinforced and over-reinforced beams is identified and marked on the graph. This comparative study has found that the concrete compressive strength has a less significant impact on the normalized ultimate flexural moment capacity for under-reinforced beams. However, it has a remarkable impact on over-reinforced beams. This characteristic is attributed to the compression-controlled behavior where the beam failure is more reliant on the concrete rectangular stress block in compression. For the under-reinforced beam, the material properties of the CFRP bar bond strength between CFRP bars to concrete govern the ultimate moment capacity of the beam itself. The concrete tensile strength in the tensile zone area is ignored [6,18,52].

### 4.4. Variation of CFRP Bars Surface Treatment

Surface treatment is an important parameter in affecting the bond strength between CFRP bars to concrete. Sufficient bond strength prevents CFRP RC beam experiences in de-bond failure as well as avoids premature slippage between CFRP bars and concrete. Generally, surface geometries can be categorized as deformed and non-deformed CFRP bars. Deformed CFRP bars consist of helically wrapped, indented and ribbed surfaces whereas, non-deformed CFRP bars have smooth or sand-coated surfaces with no geometrical ratio.

**Figure 8:**
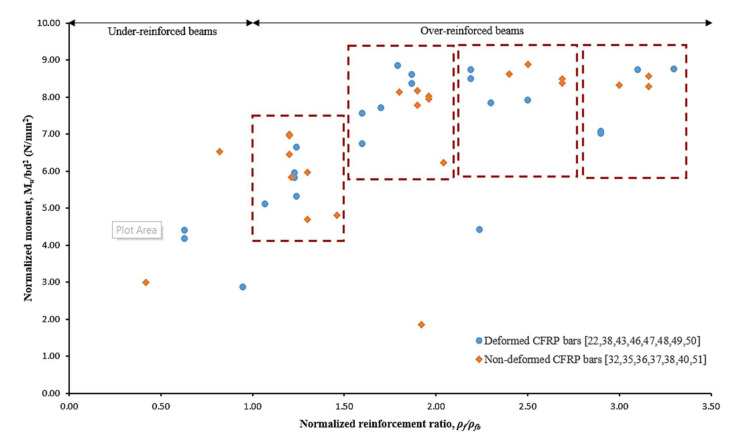
Effects of surface treatment on the normalized ultimate flexural moment capacity [22,32,35,36,37,38,40,43,46,47,48,49,50,51].

Figure 8 presents scattered data on the normalized ultimate flexural moment capacity against normalized reinforcement ratio with respect to deformed and non-deformed CFRP bars. The data pattern was grouped in the red rectangle. It can be observed that non-deformed bars require a higher reinforcement ratio to achieve about the same values of ultimate flexural strength for deformed CFRP bars. This behavior can be attributed to the variation of bond stress transferring mechanisms between CFRP bars and concrete.

#### 4.4.1. Deformed CFRP Bars

**Figure 9:**
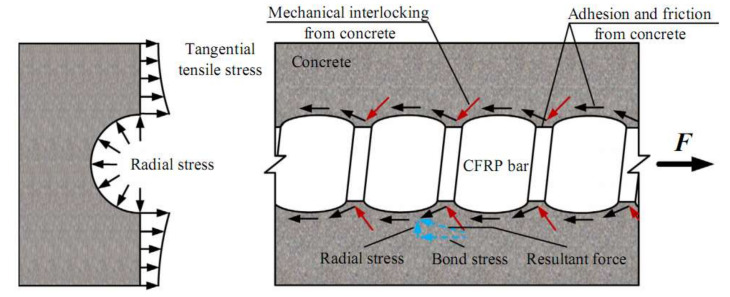
Bond stress transferring mechanisms between deformed CFRP bar and concrete (Reprinted with permission from ref. [41], 2022, Elsevier).

The bond stress transferring mechanisms between deformed CFRP bar and concrete are well described by Pour et al. [53] and Zhang et al. [41], as illustrated in Figure 9. The frictional resistance, chemical adhesion and mechanical interactions of CFRP bar to the concrete are governed by deformability of bar surface geometries that are represented by geometrical ratios [54,55]. Three geometrical ratios have been highlighted by Baena et al. [56], Okelo & Yuan [57] and Hao et al. [55] pertaining to the influence of deformed CFRP bar geometries on bond behavior with concrete: (i) area to space ratio (*a_s_*); (ii) concrete lug ratio (*CLR*); and, (iii) the relative rib area (*R_r_*).

**Figure 10:**
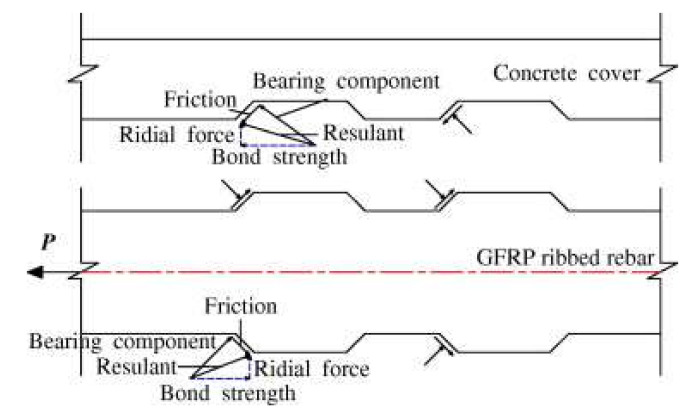
Geometrical components for deformed CFRP bar (Reprinted with permission from ref. [55], 2022, Elsevier).

All three geometrical ratios are presented in Figure 10 and can be calculated by using Equation (11)–(13), respectively.

(11) $$a_{s}=\frac{A_{r}}{r_{s}}$$

(12) $$R_{r}=\frac{A_{r}}{PR_{s}}$$

(13) $$CLR=\frac{W_{c}}{W_{c}+W_{f}}$$

where *A_r_* is the projected rib area (mm^2^); *r_s_* is the rib spacing (mm); *P* is the nominal bar perimeter (mm); *w_c_* is the concrete lug width (mm); and, *w_f_* is the CFRP bar lug width (mm).

According to the investigation by Hao et al. [55], design recommendations have been suggested on the optimal surface geometries of the deformed FRP bar. They have concluded that a deformed FRP bar with rib spacing equal to bar diameter and rib height is 6% of the bar diameter, and resulted in the best bond strength with concrete. The *R_r_* value for this FRP is equal to 0.06 [55].

#### 4.4.2. Non-Deformed CFRP Bars

Analogously, the load transferring mechanisms for non-deformed CFRP bar are provided by the only frictional resistance and chemical adhesion. In this regard, bond strength is strongly dependent on the transverse pressure from concrete due to the absence of geometrical ratios [56]. Concerning geometrical ratios, CFRP bar with smooth surfaces, however, are not recommended for structural applications unless sufficient anchoring elements are provided [58]. As for CFRP bars with sand coating, the sand coating surface may start to de-bond from the CFRP bar after reaching its maximum bond strength [56].

### 4.5. Deflection Characteristics

**Figure 11:**
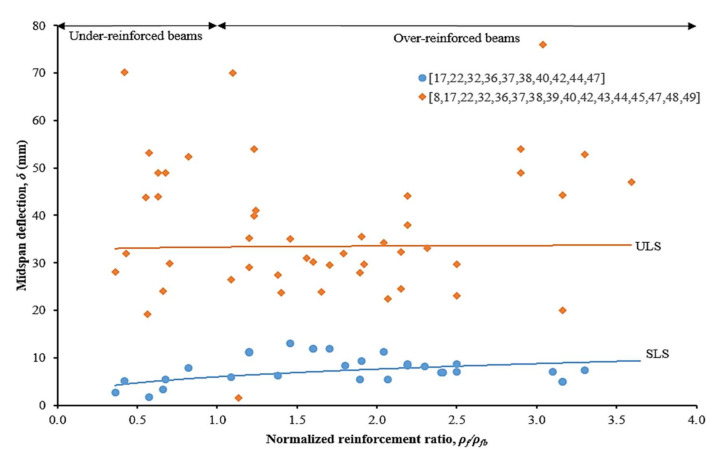
Effects of increasing normalized reinforcement ratio to mid-span beam deflections [8,17,22,32,33,34,35,36,37,38,39,40,42,43,44,45,46,47,48,49].

Generally, two stages of deflection behavior were observed for the CFRP RC beam. The first one is a linear section with a steep slope, which is corresponding to an un-cracked beam condition. This stage is known as service limit state (SLS). After achieving the cracking load, the slope drops due to the progressive cracks on the beam. The second stage is the settling the cracking process, in which a nearly linear section was recorded until the beam failure. The beam failure is recognized in excessive deflection at that particular load. The second stage is called ultimate limit state (ULS). Figure 11 shows the effects of increasing normalized reinforcement ratio to mid-span beam deflections. By increasing the reinforcement ratio from under-reinforced to over-reinforce of CFRP RC beams, there are no significant changes in the mid-span deflection at SLS. The average mid-span values were recorded at 4.41 mm and 8.51 for under-reinforced and over-reinforced beams, respectively. This behavior indicated that mid-span deflection at SLS is depending on the concrete tensile strength and CFRP bar‘s modulus of elasticity.

On the other hand, by increasing the reinforcement ratio, the deflection is decreased in mid-span deflection at ULS [17,38]. The average mid-span values were recorded at 28.23 mm and 37.47 for under-reinforced and over-reinforced beams, respectively. At ULS, deflection behavior is attributed to the high reinforcement area in resisting the applied load. In addition, mid-span deflection recovery was observed in over-reinforced CFRP RC beams after the load was released [22]. This phenomenon indicated that CFRP bars remained within the elastic behavior at the ULS stage particularly for *ρ_f_* /*ρ_fb_* ≥ 1.4. Nonetheless, a thorough understanding of the mid-span deflections behavior of CFRP RC beams from SLS to ULS is still limited in order to present the actual structural response [7].

### 4.6. Crack Width, Crack Spacing and Crack Number

Excessive deformation and deflection of CFRP RC beams will result in inducing flexural cracks. The flexural cracks are formed when the principal tensile stress exceeds the concrete tensile strength [37]. The flexural cracks propagation is perpendicular to the direction of high local tensile stress. Extensive cracks in terms of spacing and width may affect the flexural behavior of the beam, including the subsequent neutral axis may immediately shift into the compression zone after cracking [22,59].

**Figure 12:**
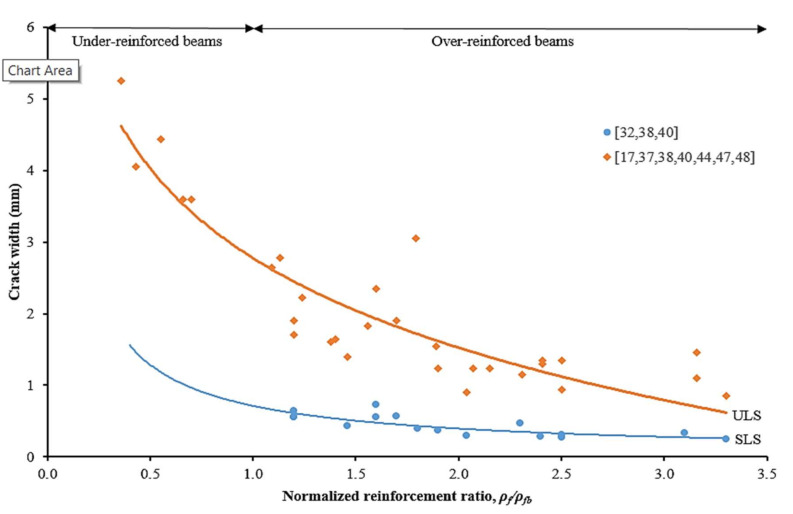
Relationships between crack width and normalized reinforcement ratio at SLS and ULS [17,32,37,38,40,44,47,48].

Figure 12 shows the relationships between crack width and normalized reinforcement ratio at SLS and ULS, respectively. It can be noticed that higher crack width was observed for the beam with a low reinforcement ratio. Data mined from literature had recorded that the average crack widths were 0.44 mm for over-reinforced beams at SLS. Whilst, at ULS, the average crack widths were 4.19 mm and 1.63 mm for under-reinforced and over-reinforced beams, respectively. The experimental results observed that over-reinforced beams with a large number of cracks resulted in low values of crack width [17,32,37,38,40,47]. Over-reinforced beams were also recorded to have a slower rate in crack development speed [11,40]. A study by Rafi et al. [22] stated that these cracks may close when the load is removed. This phenomenon indicated that CFRP bars exhibit linearly elastic behavior with sufficient bond strength of CFRP bars to concrete.

In addition to that, crack spacing at beam failure could be compared with the spacing of the stirrups [59]. The crack width and spacing at ULS might also have indicated the good interaction between materials and sufficient bond strength between CFRP bar and concrete.

On the other hand, splitting cracks along the reinforcement could appear on the concrete beam prior to flexural cracks. This type of crack was induced due to bond and anchorage failure between the reinforcement and concrete [40,59]. Hence, it should be avoided anyway in order for CFRP RC beams to achieve their ultimate flexural strength capacity.

## 5. Theoretical Prediction of Flexural Moment Capacity

In this study, the theoretical ultimate moment, *M_u_* of CFRP RC beams were calculated based on the equations provided by ACI 440.1R-15 [6], CSA S806-12 [18] and as a proposed method by Kara and Ashour [25], then compared with the experimental ultimate moment capacity, *M_u, exp_* from literature.

### 5.1. International Design Provisions

The design philosophy for the CFRP RC beams as guided by ACI 440.1R-15 [6] and CSA S806-12 [18] are based on the types of beam flexural failure modes as discussed in Section 3.2. These guidelines considered several assumptions on computing the ultimate flexural strength of CFRP RC beams, as summarized in Table 1.

On the basis of ACI 440.1R-15 [6], the ultimate flexural moment for over-reinforced beams (i.e., *ρ_f_* > *ρ_fb_*) can be derived based on the equilibrium of forces and strain compatibility as seen in Equation (14):

(14) $$M_{u-OR,\;ACI}=\rho_{f}f_{f}\left(1-0.59\frac{\rho_{f}f_{f}}{f_{c}^{\prime }}\right)bd^{2}$$

where *b* is the width of the rectangular cross-section (mm); *d* is the distance from extreme compression fiber to centroid of tension reinforcement (mm); and, $f_{f}$ is the stress level in CFRP bar (MPa) that can be calculated as follows:

(15) $$f_{f}=\left(\sqrt{\frac{{\left(E_{f}\varepsilon_{cu}\right)}^{2}}{4}+\frac{0.85\beta_{1}f_{c}^{\prime }}{\rho_{f}}E_{f}\varepsilon_{cu}}-0.5E_{f}\varepsilon_{cu}\right)\leq f_{fu}$$

On the other hand, the ultimate flexural moment for under-reinforced CFRP RC beams (i.e., *ρ_f_* ≤ *ρ_fb_*) can be calculated by using Equation (16):

(16) $$M_{u-UR,\;ACI}=A_{f}f_{fu}\left(d-\frac{\beta_{1}c}{2}\right)$$

where *c* is the distance from extreme compression fiber to the neutral axis (mm) and can be calculated in the following equation:

(17) $$c=\frac{A_{f}f_{f}}{0.85f_{c}^{\prime }b\beta_{1}}$$

Secondly, CSA S806-12 [18] considers concrete crushing as the only controlling limit state on the CFRP RC beam. Hence, the beam is designed as an over-reinforced section and the ultimate flexural moment can be calculated using Equation (18).

(18) $$M_{u-OR,\;CSA}=\rho_{f}f_{f}bd^{2}\left(1-\frac{\rho_{f}f_{f}}{2\alpha_{1}f_{c}^{\prime }}\right)$$

where CFRP bar stress, $f_{f}$ (MPa) and neutral axis, *c* (mm) can be determined by the following equations:

(19) $$f_{f}=A_{f}E_{f}\frac{\varepsilon_{cu}\left(d-c\right)}{c}<f_{fu}$$

(20) $$\alpha_{1}\beta_{1}f_{c}^{\prime }bc-A_{f}E_{f}\frac{\varepsilon_{cu}\left(d-c\right)}{c}=0$$

### 5.2. Proposed Method by Kara and Ashour

**Figure 13:**
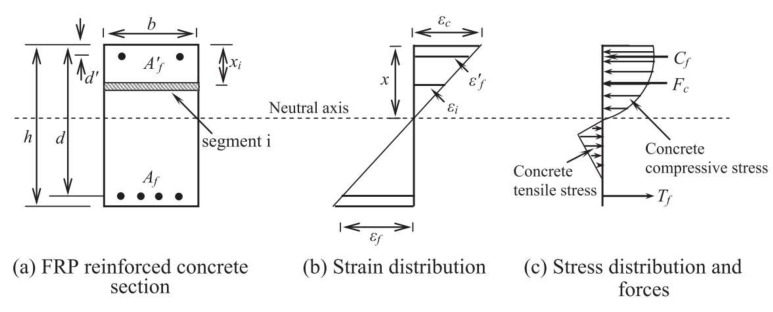
Strains, stresses and forces of FRP RC beam section (Reprinted with permission from ref. [25], 2022, Elsevier.

Kara and Ashour [25] proposed a method to calculate the ultimate moment, *M_u_* for the FRP RC beam by dividing the beam cross-section into a number of segments. Figure 13 presents a concrete section reinforced with top and bottom FRP bars. In this method, they assume that the perfect bond between concrete and FRP bars and plane section before bending remains plane after bending. Therefore, the strain in concrete and CFRP reinforcement is proportional to the distance from the neutral axis as depicted in Figure 13b, and can be expressed as below:

(21) $$\varepsilon_{f}^{\prime }=\frac{x-d^{\prime }}{x}\varepsilon_{c}$$

(22) $$\varepsilon_{f}=\frac{x-d}{x}\varepsilon_{c}$$

where $\varepsilon_{f}$ and $\varepsilon_{f}^{\prime }$ indicate the strains in the bottom and top FRP bars, respectively, *x* (mm) is a neutral axis depth, *d* and *d′* are the bottom and top FRP reinforcement depths (mm), respectively, and $\varepsilon_{c}$ is the strain at the top compression level of the RC section.

The total concrete force can be calculated with the summation of all number of segments and can be expressed in Equation (23). This summation extends over all compressive and tensile concrete forces. The compressive and tensile concrete forces can be determined from the respective stress–strain relationships as discussed in Section 2.

(23) $$F_{c}=\sum_{i=1}^{n}f_{ci}h_{i}b$$

where $f_{ci}$ (MPa) is the concrete compressive or tensile stress at the centroid of the *i*-th segment, $h_{i}$ = h/n (mm) is the thickness of the *i*-th segment and *b* (mm) is the beam width. The corresponding forces for top and bottom FRP bars are calculated from Equations (24) and (25):

(24) $$T_{f}=A_{f}E_{f}\varepsilon_{f}$$

(25) $$C_{f}=A_{f}^{\prime }E_{f}^{\prime }\varepsilon_{f}^{\prime }$$

where $T_{f}$, $A_{f}$, and $E_{f}$ are the force (kN), area (mm^2^), and modulus of elasticity of bottom FRP bars (MPa), respectively, and $C_{f}$, $A_{f}^{\prime }$, and $E_{f}^{\prime }$ are the values for compression FRP or steel reinforcement. By considering the forces equilibrium in Figure 13c, the following equation can be derived:

(26) $$F_{c}+C_{f}=T_{f}$$

(27) $$\sum_{i=1}^{n}f_{ci}h_{i}b+A_{f}^{\prime }E_{f}^{\prime }\varepsilon_{f}^{\prime }=A_{f}E_{f}\varepsilon_{f}$$

The neutral axis depth, *x* is the only unknown parameter resulting from Equation (13). Hence, the *x* value is iteratively adjusted by using the bisection method. The ultimate moment, *M_u_* for FRP RC beam is calculated by taking moments of internal forces about the neutral axis, resulting in the equation below:

(28) $$M_{u}=\sum_{i=1}^{n}F_{ci}\left(x-x_{i}\right)+T_{f}\left(x-d\right)+C_{f}\left(x-d^{\prime }\right)$$

### 5.3. Comparison between Predicted Ultimate Normalized Moment Capacities against Experimental Values

**Figure 14:**
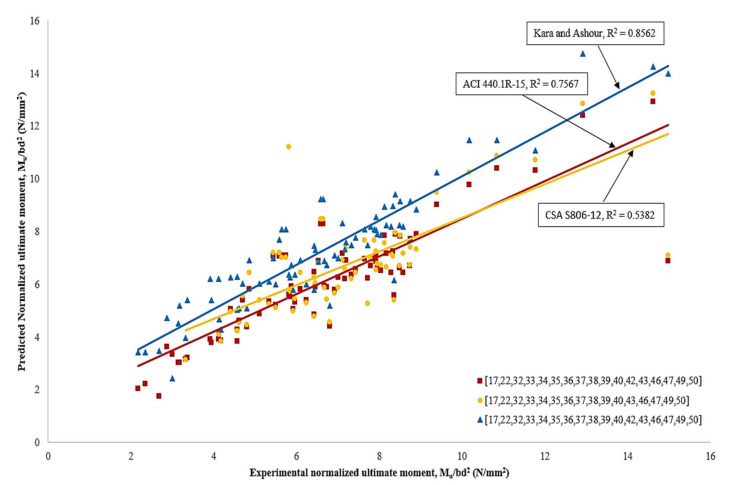
Comparison on the predictions for ultimate normalized moment capacities against experimental moment capacities [6,17,18,22,32,33,34,35,36,37,38,39,40,42,43,46,47,49,50].

Figure 14 compares the predictions for ultimate normalized moment capacities between international guidelines and as proposed by Kara and Ashour [25] against the experimental moment capacities. It can be observed that prediction values obtained from ACI 440.1R-15 [6], CSA S806-12 [18] and Kara and Ashour [25] are in very good agreement with the experimental results. The coefficient of determination (R^2^) for these relationships is 0.76, 0.54 and 0.86, respectively. In general, ACI 440.1R-15 [6] and CSA S806-12 [18] predictions have mostly underestimated the ultimate moment capacity for CFRP RC beams. Such a conclusion was the same as reported by Ahmed et al. [17], Ashour and Family [36] and Kara and Ashour [25]. This can be attributed to the ACI 440.1R-15 [6] and CSA S806-12 [18] equations that ignore the concrete tensile strength and reinforcement in the compression zone.

On the other hand, Kara and Ashour [25] predictions are more accurate with the experimental values. This can be attributed to the fact that Kara and Ashour [25] has considered the concrete tensile strength and reinforcement in the compression zone in their equation. However, the absentee factors of $\alpha_{1}$ and $\beta_{1}$ could affect the concrete compressive stress values. In addition, the difference in considering concrete compressive strain values and the assumption on perfect bond for all types of FRP with concrete may affect the final results.

## 6. Hotspot Research Topics for Future Investigations

Based on this comprehensive review, several hotspot research topics are highlighted. Further considerations are recommended for future studies by researchers worldwide, as follows:

- The bond strength between CFRP bar and concrete: Bond mechanisms between CFRP bar and concrete contribute significant roles in transferring flexural stresses in the tension zone of CFRP RC beams [8]. The sufficient bond strength of CFRP bar to concrete may reduce the crack width and deflection, subsequently increasing the beam stiffness [60]. In the extensive investigation by researchers, they have concluded that CFRP bar’s surface treatment plays enormous roles in affecting various bond strengths [41,58,61,62,63]. Therefore, applying CFRP bar with different surface treatments as flexural reinforcement could have resulted in various bond strengths. Concerning this, extensive investigation on the local bond-slip relationship in CFRP RC beam may become significant to avoid premature slip or de-bond failure of CFRP bars from concrete [42]. Henceforth, perfect bond strength between CFRP bar and concrete is assumed by international guidelines and researchers [5,6,18,19,25,32,40].
- Strength limit state design (under-reinforced CFRP RC beam): It is noteworthy that most CFRP RC beams were designed with an over-reinforced limit state. The compression failure was more desirable due to its less violent than tension failure [7,19]. However, failure of the over-reinforced CFRP RC beam is reliant on the concrete compressive strength itself. The advantages of CFRP bar particularly having high tensile strength may become less significant. The CFRP bar has never reached its ultimate tensile strength for an over-reinforced CFRP RC beam, therefore, it is necessary to consider CFRP RC beams as designed with an under-reinforced limit state. Thorough investigations on the CFRP RC beam behavior at SLS and ULS need to be conducted by taking into account that the large deflection and crack width are within tolerance.
- Application of CFRP bars in geopolymer concrete: Henceforth, few studies have been conducted to investigate the behavior of CFRP bars as flexural reinforcement in geopolymer concrete. The combination of both materials might provide high performance and sustainable structures. A study by Ahmed et al. [17] has discovered the high potential for the application of these materials. Parameters, such as reinforcement ratio and geopolymer compressive strength had affected the flexural strength, deflection, crack numbers, crack width as well as stiffness of the beam specimens. They also noticed the de-bond failure of CFRP bars from the concrete. However, investigation on the shear strength and behavior in future studies might as well contribute to the extending of knowledge.
- Application of CFRP bars in precast concrete beam: Precast concrete structures nowadays are the preferable construction method due to their cost, quality and time effectiveness in completing such projects. The application of CFRP bars as flexural reinforcement in the precast concrete beam may enhance the performance and durability of the beam itself against an aggressive environment. It is important to study the application of CFRP bars in the precast concrete beam due to the following reasons: (i) Construction in the precast industries involve the highest quality control. Handling CFRP bars in the construction operations should be performed in a professional manner to minimize damages to the bars. CFRP bars should be handled, stored and placed accordingly; and, (ii) high strength concrete in the precast beam allows for a better combination of high-strength properties of CFRP bars. In addition, precast concrete beams are non-rectangular in shape and their flexural and shear behavior have yet to be confirmed by experimental results [6].

## 7. Conclusions

On the basis of performing the analysis of the systematic literature, several conclusions can be manifested with regard to flexural strength of CFRP RC beams:

- CFRP RC beam experiences four different types of failure modes. The first one is tension failure for under-reinforced beam, followed by tension-compression and compression failure for over-reinforced beam. In addition, another failure mode that has to be considered is the de-bonding of CFRP bars from surrounding concrete.
- Over 98 rectangular CFRP RC beam specimens were mined from the works of literature and presented a comprehensive overview from the systematic review analysis. Primary factors that affect the flexural strength of CFRP RC beams were identified and quantitatively plotted to fully understand their aspect behaviors. Specifically, a factor such as reinforcement ratio has an enormous impact on the behavior and stiffness of the beam specimens for cracking and ultimate moment capacity. Another primary factor, such as concrete compressive strength had resulted in a different significant impact on CFRP RC beams pertaining to their strength limit state design. Moreover, the CFRP bar’s surface treatment plays a vital role in transferring flexural stress in the CFRP RC beam tension zone. Sufficient bond strength between CFRP bars and concrete contribute to a significant role in achieving the ultimate moment capacity of the CFRP RC beam. The combination of these primary factors has ensured the excellent performances of the CFRP RC beam at both SLS and ULS.
- Predictions on the ultimate flexural moment capacity have been specified in the international design standards and as proposed by Kara and Ashour. They are summarized to ensure a better understanding of their capability. The predictions on the ultimate flexural moment capacity obtained from Kara and Ashour have more accurate results with the experimental values compared to ACI 440.1R-15, CSA S806-12.

## Figures and Tables

**Table 1:** Assumptions made on CFRP RC beams by international standards and current research.

Compartment	ACI 440.1R-15 [6]	CSA S806-12 [18]	Kara and Ashour [25]
Strain	Strain in concrete and CFRP reinforcement is proportional to the distance from the neutral axis		
Concrete compressive strain	0.0030	0.0035	0.0035
Concrete tensile strength	Ignored	Ignored	Accounted
Bond strength of CFRP to concrete	Perfect bond	Perfect bond	Perfect bond
Strength limit state	Over-reinforced and under-reinforced	Over-reinforced	Over-reinforced and under-reinforced

## Data Availability

Data sharing is not applicable.
